# Supplementary material for: A deep learning approach for enhancing pandemic prediction: A retrospective evaluation of transformer neural networks and multi-source data fusion for infectious disease forecasting
Source: Epidemics. Author manuscript; Available in PMC 2025 Dec 12. (PMC12696886; doi:10.1016/j.epidem.2025.100865)
Supplement: Appendix Tables and Figures [file NIHMS2127972-supplement-Appendix_Tables_and_Figures.pdf]

Table A1. County-Level Percentage Agreement (PA %) and interquartile range (IQR) for daily prediction.

			Full Model with X Input	Full Model with no X Input	Persistence model	ARIMA	Full Model with X Input	Full Model with no X Input	Persistence model	ARIMA
			Case				Death			
Day 1	12/01/2021 -	Median	86.15	79.56	85.47	73.36	81.25	82.1	84.98	82.98
	3/31/22	(Q1; Q3)	(84.92; 88.39)	(77.79; 81.09)	(83.11; 87.12)	(69.76; 79.06)	(73.92; 86.45)	(69.91; 85.86)	(75.01; 88.83)	(77.84; 86.02)
	05/01/2022 -	Median	77.64	81.06	76.04	74.74	58.47	57.64	59.42	78.63
	10/31/22	(Q1; Q3)	(72.7; 79.91)	(76.98; 82.91)	(71.75; 79.29)	(70.36; 78.91)	(41.9; 68.08)	(37.16; 69.8)	(40; 70.65)	(65.27; 81.38)
	12/01/2022 -	Median	78.59	81.5	77.97	75.56	70.92	67.62	68.82	82.81
	2/28/23	(Q1; Q3)	(76.24; 81.82)	(77.39; 83.44)	(74.87; 80.6)	(72.37; 79.39)	(40; 79.28)	(41.67; 76.18)	(41.67; 79.25)	(70.64; 87.04)
Day 2	12/01/2021 -	Median	84.16	76.16	78.78	58.03	84	83.2	82.84	72.32
	3/31/22	(Q1; Q3)	(82.41; 86.75)	(74.75; 78.05)	(76.5; 80.2)	(52.22; 62.64)	(75.17; 87.5)	(70.28; 86.29)	(72.17; 87)	(63.02; 76.09)
	05/01/2022 -	Median	75.59	74.65	76.04	63.45	56.62	58.22	59.42	65.01
	10/31/22	(Q1; Q3)	(69.76; 78.29)	(69.54; 77.25)	(71.75; 79.29)	(58.72; 67.21)	(40; 70.31)	(40; 70.19)	(40; 70.65)	(52.44; 68.92)
	12/01/2022 -	Median	77.47	75.16	77.97	66.06	68.06	67.72	68.82	70.49
	2/28/23	(Q1; Q3)	(73.87; 79.89)	(72.33; 77.5)	(74.87; 80.6)	(58.65; 70.28)	(40; 76.67)	(39.65; 79.05)	(41.67; 79.25)	(59.16; 77.09)
Day 3	12/01/2021 -	Median	83.1	75.89	73.46	46.84	85.59	82.84	80.99	62.44
	3/31/22	(Q1; Q3)	(80.76; 85.36)	(74.18; 77.61)	(71; 75.47)	(42.85; 50.66)	(76.88; 89.13)	(71.56; 86.35)	(69.31; 85.5)	(54.57; 68.50)
	05/01/2022 -	Median	74.96	74.78	76.04	57.64	59.99	58.84	60.04	55.84
	10/31/22	(Q1; Q3)	(69.96; 78.23)	(69.89; 77.66)	(71.75; 79.29)	(55.15; 61.96)	(36.37; 71.21)	(35.83; 69.87)	(40; 70.65)	(47.47; 61.67)
	12/01/2022 -	Median	77.7	76.24	76.23	61.2	71.15	67.88	68.06	63.64
	2/28/23	(Q1; Q3)	(74.06; 80.04)	(72.5; 78.18)	(73.22; 78.25)	(56.96; 66.33)	(33.33; 79.11)	(41.67; 77.82)	(41.67; 78.8)	(52.92; 71.25)
Day 4	12/01/2021 -	Median	81.38	75.84	70.33	37.78	84.15	82.26	80.03	55.99
	3/31/22	(Q1; Q3)	(79.49; 82.52)	(73.08; 77.88)	(67.8; 71.97)	(34.44; 40.60)	(74.48; 88.7)	(70.65; 85.8)	(67.36; 84.71)	(50.34; 61.24)
	05/01/2022 -	Median	75.79	72.83	74.89	51.92	60.01	59.75	58	51.11
	10/31/22	(Q1; Q3)	(72.18; 78.57)	(68.34; 76.46)	(69.93; 78.09)	(49.82; 57.18)	(36.73; 70.59)	(40; 69.83)	(43.33; 68.54)	(41.49; 55.52)
	12/01/2022 -	Median	76.42	75.89	76.23	59.73	70.64	67.9	68.06	57.97
	2/28/23	(Q1; Q3)	(73.41; 78.32)	(72.13; 78.93)	(73.22; 78.25)	(55.46; 62.87)	(33.33; 80.89)	(41.67; 76.93)	(41.67; 78.8)	(47.28; 69.12)
Day 5	12/01/2021 -	Median	79.39	75.65	67.47	31.38	83.86	81.88	77.75	52.37
	3/31/22	(Q1; Q3)	(78.12; 80.64)	(73.38; 77.75)	(64.72; 68.85)	(26.79; 33.50)	(74.63; 87.26)	(71.59; 85.72)	(67.48; 83.85)	(47.00; 58.63)

	05/01/2022 -	Median	75.06	74.29	74.89	48.46	58.41	59.2	58	46.71
	10/31/22	(Q1; Q3)	(70.81; 77.41)	(69.26; 76.94)	(69.93; 78.09)	(45.24; 53.80)	(36.53; 70.08)	(40; 70.78)	(43.33; 68.54)	(36.70; 50.88)
	12/01/2022 -	Median	74.89	75.75	76.23	55.13	68.33	67.85	68.06	55.39
	2/28/23	(Q1; Q3)	(71.28; 76.99)	(72.53; 78.68)	(73.22; 78.25)	(51.48; 61.34)	(40; 79.52)	(41.67; 76.85)	(44.44; 78.8)	(42.43; 64.59)
**Day 6**	12/01/2021 -	Median	84.65	79.89	64.3	26.93	82.1	79.03	76.9	48.94
	3/31/22	(Q1; Q3)	(83.39; 86.92)	(77.95; 82.01)	(61.42; 65.91)	(24.41; 29.15)	(71.92; 86.87)	(65.47; 84.56)	(66.18; 82.59)	(43.51; 55.51)
	05/01/2022 -	Median	74.91	74.58	75.54	45.09	59.36	56.93	59.42	43.93
	10/31/22	(Q1; Q3)	(70.12; 79.02)	(69.77; 78.32)	(68.86; 77.85)	(41.43; 49.20)	(41.79; 68.21)	(38.75; 68.26)	(40.25; 68.09)	(33.50; 48.00)
	12/01/2022 -	Median	77.38	77.06	76.23	55.17	66.65	62.7	68.06	53.53
	2/28/23	(Q1; Q3)	(74.63; 80)	(74.47; 79.72)	(73.22; 78.25)	(51.64; 59.39)	(40; 75.92)	(41.67; 73.09)	(44.44; 78.8)	(41.24; 62.70)
**Day 7**	12/01/2021 -	Median	84.31	77.89	60.25	24.62	82.36	78.66	74.67	47.35
	3/31/22	(Q1; Q3)	(82.95; 86.78)	(76.3; 79.88)	(57.45; 62.04)	(22.04; 28.00)	(71.52; 86.68)	(65.07; 83.48)	(65.18; 81.23)	(39.15; 52.60)
	05/01/2022 -	Median	74.66	72.13	73.02	41.97	58.45	56.73	59.42	41.36
	10/31/22	(Q1; Q3)	(70; 78.84)	(67.02; 75.09)	(67.32; 76.64)	(38.61; 46.42)	(40; 69.05)	(38.75; 65.63)	(40.25; 67.56)	(30.41; 47.66)
	12/01/2022 -	Median	77.47	74.83	72.58	53.06	66.65	61.6	67.32	51.44
	2/28/23	(Q1; Q3)	(74.31; 79.85)	(72.19; 76.79)	(68.97; 75.12)	(48.48; 56.57)	(40; 76.37)	(41.67; 71.38)	(44.44; 77.87)	(39.85; 61.00)

Table A2. County-Level Percentage Agreement (PA %) and interquartile range (IQR) for weekly prediction.

			Full Model with X Input	Full Model with no X Input	Persistence model	ARIMA	Full Model with X Input	Full Model with no X Input	Persistence model	ARIMA
			Case				Death			
Week 1	12/01/2021 -	Median	73.98	64.43	64.16	70.99	84.96	83.58	83.82	82.53
	3/31/22	(Q1; Q3)	(71.36; 75.4)	(62.79; 66.25)	(62.48; 66.54)	(68.09; 74.05)	(81.49; 87.55)	(81.13; 86.07)	(81.17; 86.18)	(77.79; 86.40)
	05/01/2022 -	Median	82.59	80.43	77.08	77.75	83.2	80.39	83.3	76.17
	10/31/22	(Q1; Q3)	(78.77; 85.13)	(76.65; 83.29)	(72.4; 79.76)	(73.13; 81.74)	(80.71; 85.65)	(76.75; 82.47)	(82.02; 86.2)	(62.63; 81.35)
	12/01/2022 -	Median	81.62	78.69	76.08	76.21	86.34	81.79	84.42	80.98
	2/28/23	(Q1; Q3)	(78.85; 83.82)	(75.09; 81.23)	(73.45; 79.39)	(72.28; 80.76)	(83.25; 89.36)	(77.81; 85.12)	(78.57; 89.09)	(67.26; 86.73)
Week 2	12/01/2021 -	Median	73.34	63.88	47.48	53.56	86.42	83.6	73.67	72.65
	3/31/22	(Q1; Q3)	(71.32; 75.31)	(62.34; 65.92)	(44.77; 49.44)	(49.04; 56.79)	(83.11; 88.1)	(80.39; 86.66)	(68.7; 76.88)	(62.36; 77.16)
	05/01/2022 -	Median	82.15	78.16	67.91	65.91	82.21	83.74	72.86	61.93
	10/31/22	(Q1; Q3)	(78.1; 84.19)	(73.89; 81.07)	(63.15; 70.08)	(59.96; 70.12)	(79.42; 84.64)	(81.15; 85.62)	(68.72; 76.31)	(51.12; 68.15)
	12/01/2022 -	Median	82.01	77.13	70.62	67.03	84.54	85.93	74.02	69.68
	2/28/23	(Q1; Q3)	(79.86; 84.48)	(73.21; 79.64)	(65.96; 73.62)	(61.33; 71.53)	(80.55; 88.65)	(81.67; 88.42)	(65.31; 81.85)	(56.67; 76.48)
Week 3	12/01/2021 -	Median	72.48	64.09	36.24	41.2	87.83	83.08	65.9	63.81
	3/31/22	(Q1; Q3)	(69.64; 73.96)	(62.2; 65.98)	(34.06; 39.51)	(38.37; 44.36)	(84.78; 89.4)	(79.77; 85.48)	(59.31; 69.4)	(54.73; 68.67)
	05/01/2022 -	Median	81.2	76.94	59.34	58.94	85.46	82.42	63.31	53.82
	10/31/22	(Q1; Q3)	(76.66; 83.3)	(72.36; 79.62)	(56.99; 62.96)	(55.14; 62.80)	(83.96; 87.02)	(80.09; 85.17)	(57.41; 69.87)	(44.48; 60.05)
	12/01/2022 -	Median	81.69	76	62.7	60.98	86.52	84.37	65.09	64.85
	2/28/23	(Q1; Q3)	(79.67; 84.56)	(73.24; 79.46)	(58.94; 66.2)	(55.85; 66.23)	(83.33; 89.36)	(79.23; 87.22)	(57.14; 75.94)	(50.18; 72.20)
Week 4	12/01/2021 -	Median	70.32	64.06	30.52	32.14	87.66	82.22	58.97	57.65
	3/31/22	(Q1; Q3)	(68.96; 72.48)	(61.54; 65.81)	(28.55; 33.01)	(29.14; 34.74)	(84.65; 89.78)	(78.81; 84.86)	(51.58; 63.42)	(49.75; 61.08)
	05/01/2022 -	Median	80.95	73.53	54.23	53.91	86.51	80.15	54.94	49.95
	10/31/22	(Q1; Q3)	(76.98; 82.42)	(68.99; 76.16)	(50.9; 57.14)	(50.16; 57.02)	(84.64; 88.41)	(77.8; 83.17)	(50.92; 65.25)	(38.84; 55.44)
	12/01/2022 -	Median	80.34	74.72	58.58	58.98	88.02	81.76	60.05	59.53
	2/28/23	(Q1; Q3)	(78.2; 82.66)	(71.12; 78.4)	(54.34; 62.86)	(57.01; 61.63)	(85.01; 91.42)	(77.11; 85.04)	(47.98; 71.33)	(44.86; 67.63)
Week 5	12/01/2021 -	Median	68.72	63.02	26.85	25.38	86.84	82.75	53	53.13
	3/31/22	(Q1; Q3)	(66.66; 71)	(61.19; 65.55)	(24.95; 29.08)	(22.32; 28.30)	(84.17; 88.6)	(79.59; 85.04)	(46.72; 59.54)	(47.45; 59.37)

05/01/2022 -	Median	79.6	72.94	49.45	49.62	84.35	82.63	48.72	45.87
10/31/22	(Q1; Q3)	(75.48; 81.73)	(68.66; 75.27)	(44.94; 52.05)	(45.89; 53.16)	(82.78; 87.07)	(79.51; 84.43)	(43.27; 60.43)	(33.62; 50.92)
12/01/2022 -	Median	78.34	73.35	55.2	55.79	87.23	83.5	57.15	56.02
2/28/23	(Q1; Q3)	(74.66; 79.79)	(69.99; 77.08)	(50.34; 59)	(52.24; 58.79)	(83.58; 90.01)	(79.09; 87.26)	(44.46; 69.72)	(42.03; 64.82)

Table A3. County-Level Root Mean Square Error (RMSE) and interquartile range (IQR) for daily prediction.

			Full Model with X Input	Full Model with no X Input	Persistence model	ARIMA	Full Model with X Input	Full Model with no X Input	Persistence model	ARIMA
			Case				Death			
Day 1	12/01/2021 -	Median	7.6	14.43	8.39	7.43	0.05	0.08	0.04	0.03
	3/31/22	(Q1; Q3)	(3.27; 17.24)	(6.38; 35.04)	(3.86; 19.5)	(3.52; 17.76)	(0.03; 0.1)	(0.04; 0.14)	(0.02; 0.07)	(0.01; 0.04)
	05/01/2022 -	Median	2.91	3.18	2.68	1.8	0.02	0.03	0.01	0.01
	10/31/22	(Q1; Q3)	(0.94; 4.19)	(1.08; 6.4)	(0.88; 4.05)	(0.52; 2.76)	(0.01; 0.03)	(0.02; 0.04)	(0.01; 0.02)	(0.01; 0.03)
	12/01/2022 -	Median	1.61	1.77	1.46	0.76	0.01	0.02	0.01	0.01
	2/28/23	(Q1; Q3)	(0.87; 4.28)	(0.96; 4.42)	(0.78; 3.8)	(0.46; 2.27)	(0.01; 0.03)	(0.01; 0.04)	(0.01; 0.02)	(0.00; 0.01)
Day 2	12/01/2021 -	Median	10.01	15.54	13.19	17.48	0.05	0.07	0.06	0.05
	3/31/22	(Q1; Q3)	(4.64; 22.15)	(7.2; 37.53)	(6.27; 31.93)	(8.06; 47.86)	(0.02; 0.09)	(0.03; 0.12)	(0.03; 0.11)	(0.03; 0.08)
	05/01/2022 -	Median	3.24	4.13	3.8	2.98	0.02	0.03	0.02	0.02
	10/31/22	(Q1; Q3)	(0.97; 4.65)	(1.45; 7.92)	(1.23; 5.72)	(0.89; 4.74)	(0.01; 0.02)	(0.01; 0.03)	(0.01; 0.03)	(0.02; 0.05)
	12/01/2022 -	Median	1.59	2.27	2.05	1.27	0.01	0.02	0.01	0.01
	2/28/23	(Q1; Q3)	(0.86; 4.37)	(1.21; 5.96)	(1.1; 5.15)	(0.70; 3.60)	(0.01; 0.02)	(0.01; 0.04)	(0.01; 0.03)	(0.01; 0.02)
Day 3	12/01/2021 -	Median	10.77	16.14	17	26.67	0.05	0.07	0.08	0.07
	3/31/22	(Q1; Q3)	(5.06; 24.27)	(7.7; 37.98)	(8.33; 42.07)	(12.52; 80.41)	(0.02; 0.09)	(0.03; 0.12)	(0.03; 0.13)	(0.04; 0.11)
	05/01/2022 -	Median	3.1	4.6	4.64	3.37	0.02	0.02	0.03	0.03
	10/31/22	(Q1; Q3)	(0.94; 4.44)	(1.57; 6.85)	(1.52; 7)	(1.19; 5.70)	(0.01; 0.03)	(0.01; 0.03)	(0.01; 0.04)	(0.02; 0.06)
	12/01/2022 -	Median	1.54	2.48	2.51	1.59	0.01	0.01	0.02	0.01
	2/28/23	(Q1; Q3)	(0.83; 4.18)	(1.31; 6.36)	(1.36; 6.26)	(0.88; 4.31)	(0.01; 0.03)	(0.01; 0.03)	(0.01; 0.04)	(0.01; 0.02)
Day 4	12/01/2021 -	Median	11.14	15.18	21.03	34.5	0.05	0.07	0.09	0.08
	3/31/22	(Q1; Q3)	(5.21; 28.14)	(7.64; 36.7)	(10.13; 51.99)	(15.45; 105.32)	(0.02; 0.09)	(0.03; 0.11)	(0.04; 0.16)	(0.04; 0.15)
	05/01/2022 -	Median	3.4	4.66	5.36	3.69	0.02	0.02	0.03	0.04
	10/31/22	(Q1; Q3)	(1; 4.85)	(1.55; 6.76)	(1.75; 8.09)	(1.35; 6.52)	(0.01; 0.02)	(0.01; 0.03)	(0.02; 0.05)	(0.02; 0.08)
	12/01/2022 -	Median	1.82	2.5	2.91	1.91	0.01	0.01	0.02	0.01
	2/28/23	(Q1; Q3)	(0.97; 5)	(1.34; 6.27)	(1.57; 7.2)	(0.99; 5.16)	(0.01; 0.02)	(0.01; 0.04)	(0.01; 0.04)	(0.01; 0.02)
Day 5	12/01/2021 -	Median	13.26	15.49	24.65	39.62	0.06	0.07	0.1	0.09
	3/31/22	(Q1; Q3)	(6.11; 33.77)	(7.48; 37.22)	(11.87; 61.74)	(17.90; 119.74)	(0.03; 0.11)	(0.03; 0.12)	(0.04; 0.18)	(0.05; 0.19)

	05/01/2022 -	Median	4.1	4.96	6	3.84	0.02	0.02	0.03	0.04
	10/31/22	(Q1; Q3)	(1.2; 6.64)	(1.63; 7.14)	(1.96; 9.06)	(1.45; 7.87)	(0.01; 0.03)	(0.01; 0.03)	(0.02; 0.06)	(0.03; 0.09)
	12/01/2022 -	Median	2.34	2.68	3.25	2.09	0.01	0.01	0.02	0.02
	2/28/23	(Q1; Q3)	(1.23; 6.79)	(1.53; 6.87)	(1.76; 8.03)	(1.04; 5.62)	(0.01; 0.02)	(0.01; 0.04)	(0.01; 0.05)	(0.01; 0.03)
**Day 6**	12/01/2021 -	Median	9.8	13.69	28.36	42.5	0.05	0.07	0.11	0.1
	3/31/22	(Q1; Q3)	(4.43; 21.98)	(6.58; 33.56)	(13.76; 71.79)	(19.38; 127.41)	(0.02; 0.09)	(0.03; 0.13)	(0.05; 0.19)	(0.06; 0.22)
	05/01/2022 -	Median	2.87	3.84	6.53	4.19	0.02	0.02	0.04	0.05
	10/31/22	(Q1; Q3)	(0.98; 4.83)	(1.32; 6.49)	(2.12; 9.73)	(1.60; 9.02)	(0.01; 0.03)	(0.01; 0.04)	(0.02; 0.06)	(0.03; 0.10)
	12/01/2022 -	Median	1.61	2.2	3.56	2.23	0.01	0.02	0.02	0.02
	2/28/23	(Q1; Q3)	(0.84; 4.24)	(1.15; 5.76)	(1.92; 8.78)	(1.16; 5.77)	(0.01; 0.03)	(0.01; 0.04)	(0.01; 0.05)	(0.01; 0.03)
**Day 7**	12/01/2021 -	Median	10.22	16.38	32.37	44.15	0.05	0.08	0.12	0.11
	3/31/22	(Q1; Q3)	(4.74; 21.97)	(7.8; 40.11)	(15.74; 82.2)	(20.20; 132.05)	(0.03; 0.1)	(0.03; 0.14)	(0.05; 0.21)	(0.06; 0.24)
	05/01/2022 -	Median	2.91	4.61	6.86	4.41	0.02	0.02	0.04	0.05
	10/31/22	(Q1; Q3)	(0.99; 4.8)	(1.58; 8.43)	(2.27; 10.38)	(1.73; 10.01)	(0.01; 0.03)	(0.01; 0.04)	(0.02; 0.07)	(0.03; 0.11)
	12/01/2022 -	Median	1.58	2.62	3.84	2.41	0.01	0.02	0.02	0.02
	2/28/23	(Q1; Q3)	(0.84; 4.2)	(1.34; 7)	(2.06; 9.3)	(1.21; 5.96)	(0.01; 0.03)	(0.01; 0.04)	(0.01; 0.05)	(0.01; 0.04)

Table A4. County-Level Root Mean Square Error (RMSE) and interquartile range (IQR) for weekly prediction.

			Full Model with X Input	Full Model with no X Input	Persistence model	ARIMA	Full Model with X Input	Full Model with no X Input	Persistence model	ARIMA
			Case				Death			
Week 1	12/01/2021 - 3/31/22	Median	146.55	215.7	213.88	165.55	0.59	0.79	0.74	0.6
		(Q1; Q3)	(67.34; 356.04)	(103.14; 559.54)	(103.9; 556.19)	(79.86; 429.88)	(0.29; 1.18)	(0.34; 1.3)	(0.35; 1.27)	(0.30; 0.84)
	05/01/2022 - 10/31/22	Median	31.89	36.14	43.89	38.07	0.17	0.26	0.23	0.27
		(Q1; Q3)	(10.9; 51.26)	(11.3; 60.52)	(14.71; 68.35)	(11.55; 56.72)	(0.1; 0.35)	(0.14; 0.43)	(0.14; 0.41)	(0.17; 0.53)
	12/01/2022 - 2/28/23	Median	18.7	20.69	25.33	18.92	0.11	0.16	0.13	0.12
		(Q1; Q3)	(9.63; 46.65)	(11.7; 50.53)	(13.63; 60.52)	(10.63; 52.33)	(0.06; 0.26)	(0.08; 0.4)	(0.07; 0.36)	(0.08; 0.20)
Week 2	12/01/2021 - 3/31/22	Median	145.66	214.67	389.37	372.75	0.56	0.8	1.06	1.06
		(Q1; Q3)	(68.78; 374.51)	(104.43; 562.79)	(180.68; 1005.83)	(183.84; 1110.89)	(0.27; 1.05)	(0.36; 1.28)	(0.55; 2.13)	(0.52; 1.62)
	05/01/2022 - 10/31/22	Median	32.31	40.67	58.34	62.3	0.18	0.23	0.38	0.42
		(Q1; Q3)	(11.06; 50.73)	(13.62; 65.52)	(20.22; 92.57)	(19.98; 99.02)	(0.1; 0.36)	(0.13; 0.41)	(0.23; 0.88)	(0.29; 0.92)
	12/01/2022 - 2/28/23	Median	18.27	23.72	32.99	32.01	0.13	0.12	0.2	0.2
		(Q1; Q3)	(9.68; 44.81)	(12.91; 57.25)	(17.84; 77.79)	(16.92; 86.11)	(0.06; 0.29)	(0.07; 0.36)	(0.11; 0.59)	(0.13; 0.33)
Week 3	12/01/2021 - 3/31/22	Median	151.35	216.11	528.54	566.35	0.52	0.75	1.44	1.39
		(Q1; Q3)	(73.59; 390.75)	(104.9; 556.36)	(241.81; 1342.58)	(274.01; 1814.22)	(0.26; 0.94)	(0.36; 1.27)	(0.73; 2.88)	(0.70; 2.48)
	05/01/2022 - 10/31/22	Median	34.55	44.17	66.79	70.16	0.17	0.23	0.5	0.57
		(Q1; Q3)	(11.5; 51.91)	(14.8; 68.82)	(24.52; 110.12)	(25.78; 115.25)	(0.1; 0.29)	(0.12; 0.42)	(0.3; 1.11)	(0.36; 1.09)
	12/01/2022 - 2/28/23	Median	19.06	25.37	41.22	39.32	0.11	0.12	0.23	0.26
		(Q1; Q3)	(10.37; 46.02)	(13.62; 60.92)	(22.06; 94.36)	(19.35; 94.60)	(0.06; 0.24)	(0.07; 0.37)	(0.13; 0.72)	(0.16; 0.45)
Week 4	12/01/2021 - 3/31/22	Median	157.76	212.64	615.66	734.4	0.54	0.74	1.69	1.73
		(Q1; Q3)	(77.56; 426.13)	(103.3; 549.05)	(284.74; 1577.62)	(340.68; 2314.89)	(0.25; 0.94)	(0.36; 1.3)	(0.86; 3.51)	(0.88; 3.40)
	05/01/2022 - 10/31/22	Median	37.94	48.13	74.35	77.28	0.16	0.22	0.6	0.75
		(Q1; Q3)	(11.86; 54.89)	(16.32; 73.89)	(27.82; 138.24)	(29.50; 141.71)	(0.09; 0.3)	(0.12; 0.44)	(0.36; 1.28)	(0.41; 1.31)
	12/01/2022 - 2/28/23	Median	20.86	27.33	48.22	43.17	0.11	0.14	0.28	0.28
		(Q1; Q3)	(11.13; 50.6)	(14.56; 65.11)	(23.45; 117.36)	(22.78; 112.78)	(0.06; 0.26)	(0.07; 0.39)	(0.16; 0.8)	(0.19; 0.60)

Week 5	12/01/2021 - 3/31/22	Median	167.74	213.63	665.7	844.66	0.54	0.72	1.94	1.93
		(Q1; Q3)	(83.83; 462.56)	(102.74; 556.65)	(309.56; 1718.86)	(387.06; 2597.09)	(0.24; 0.96)	(0.36; 1.27)	(0.93; 4.2)	(1.04; 4.13)
	05/01/2022 - 10/31/22	Median	42.47	52.79	82.82	82.95	0.16	0.22	0.72	0.87
		(Q1; Q3)	(12.84; 61.6)	(18.12; 79.6)	(29.61; 162.66)	(31.25; 169.59)	(0.09; 0.32)	(0.13; 0.44)	(0.45; 1.51)	(0.46; 1.57)
	12/01/2022 - 2/28/23	Median	23.3	29.64	53.28	48.07	0.12	0.13	0.32	0.32
		(Q1; Q3)	(12.29; 59.46)	(16.38; 71.38)	(24.84; 127.18)	(22.96; 121.94)	(0.06; 0.27)	(0.07; 0.38)	(0.21; 0.88)	(0.21; 0.78)

Table A5. County-Level Mean Absolute Error (MAE) and interquartile range (IQR) for daily prediction.

			Full Model with X Input	Full Model with no X Input	Persistence model	ARIMA	Full Model with X Input	Full Model with no X Input	Persistence model	ARIMA
			Case				Death			
Day 1	12/01/2021 -	Median	4.04	8.2	3.62	2	0.03	0.05	0.01	0.01
	3/31/22	(Q1; Q3)	(1.77; 9.29)	(3.59; 20.49)	(1.67; 8.21)	(0.95; 4.76)	(0.01; 0.06)	(0.02; 0.1)	(0; 0.03)	(0.00; 0.01)
	05/01/2022 -	Median	1.41	1.97	0.66	0.49	0.01	0.01	0	0
	10/31/22	(Q1; Q3)	(0.53; 2.51)	(0.77; 4.11)	(0.25; 1.08)	(0.17; 0.82)	(0; 0.01)	(0.01; 0.03)	(0; 0)	(0.00; 0.01)
	12/01/2022 -	Median	1.06	1.17	0.41	0.26	0.01	0.01	0	0
	2/28/23	(Q1; Q3)	(0.53; 3.19)	(0.6; 3.35)	(0.22; 1.15)	(0.14; 0.81)	(0; 0.01)	(0; 0.04)	(0; 0)	(0.00; 0.00)
Day 2	12/01/2021 -	Median	4.85	8.62	6.74	4.94	0.02	0.04	0.03	0.01
	3/31/22	(Q1; Q3)	(2.3; 10.2)	(4.11; 21.95)	(3.09; 15.66)	(2.26; 12.86)	(0.01; 0.05)	(0.02; 0.08)	(0.01; 0.05)	(0.01; 0.03)
	05/01/2022 -	Median	1.12	2.44	1.32	0.8	0.01	0.01	0	0.01
	10/31/22	(Q1; Q3)	(0.41; 2.02)	(0.95; 5.03)	(0.5; 2.15)	(0.30; 1.59)	(0; 0.01)	(0; 0.02)	(0; 0.01)	(0.00; 0.01)
	12/01/2022 -	Median	0.71	1.49	0.81	0.45	0.01	0.01	0	0
	2/28/23	(Q1; Q3)	(0.4; 2.18)	(0.76; 4.29)	(0.44; 2.16)	(0.25; 1.30)	(0; 0.01)	(0; 0.02)	(0; 0.01)	(0.00; 0.01)
Day 3	12/01/2021 -	Median	5.3	8.66	9.69	7.86	0.03	0.04	0.04	0.02
	3/31/22	(Q1; Q3)	(2.46; 12.43)	(4.2; 21.05)	(4.46; 23.28)	(3.56; 21.36)	(0.02; 0.06)	(0.01; 0.07)	(0.01; 0.07)	(0.01; 0.04)
	05/01/2022 -	Median	1.07	2.2	1.98	0.99	0.01	0	0.01	0.01
	10/31/22	(Q1; Q3)	(0.37; 1.6)	(0.82; 4.1)	(0.75; 3.23)	(0.42; 1.94)	(0; 0.02)	(0; 0.01)	(0; 0.01)	(0.00; 0.02)
	12/01/2022 -	Median	0.58	1.44	1.23	0.56	0.01	0	0	0
	2/28/23	(Q1; Q3)	(0.32; 1.63)	(0.73; 3.78)	(0.66; 3.18)	(0.30; 1.45)	(0; 0.02)	(0; 0.02)	(0; 0.01)	(0.00; 0.01)
Day 4	12/01/2021 -	Median	5.93	8.44	12.51	10.09	0.03	0.04	0.05	0.03
	3/31/22	(Q1; Q3)	(2.71; 16.03)	(4.05; 20.82)	(5.75; 30.77)	(4.74; 29.34)	(0.01; 0.05)	(0.01; 0.07)	(0.02; 0.09)	(0.01; 0.06)
	05/01/2022 -	Median	1.57	2.2	2.64	1.14	0.01	0.01	0.01	0.01
	10/31/22	(Q1; Q3)	(0.54; 3.03)	(0.82; 4.01)	(1; 4.34)	(0.47; 2.41)	(0; 0.01)	(0; 0.01)	(0; 0.02)	(0.01; 0.02)
	12/01/2022 -	Median	1.19	1.31	1.64	0.68	0.01	0.01	0.01	0
	2/28/23	(Q1; Q3)	(0.58; 3.37)	(0.72; 3.51)	(0.87; 4.22)	(0.35; 1.70)	(0; 0.01)	(0; 0.02)	(0; 0.02)	(0.00; 0.01)
Day 5	12/01/2021 -	Median	7.5	8.96	15.22	12.36	0.04	0.04	0.06	0.03
	3/31/22	(Q1; Q3)	(3.44; 20.15)	(4.3; 22.13)	(7.11; 38.14)	(5.72; 35.88)	(0.01; 0.08)	(0.02; 0.08)	(0.02; 0.11)	(0.02; 0.07)

	05/01/2022 -	Median	2.22	2.58	3.3	1.3	0.01	0.01	0.01	0.01
	10/31/22	(Q1; Q3)	(0.8; 4.59)	(0.97; 4.31)	(1.25; 5.44)	(0.51; 3.00)	(0; 0.01)	(0; 0.02)	(0.01; 0.02)	(0.01; 0.03)
	12/01/2022 -	Median	1.84	1.67	2.05	0.78	0.01	0.01	0.01	0.01
	2/28/23	(Q1; Q3)	(0.89; 5.66)	(0.92; 4.4)	(1.09; 5.26)	(0.39; 1.93)	(0; 0.01)	(0; 0.02)	(0; 0.02)	(0.00; 0.01)
**Day 6**	12/01/2021 -	Median	5.53	8	17.86	13.48	0.04	0.05	0.07	0.04
	3/31/22	(Q1; Q3)	(2.49; 12.33)	(3.64; 18.31)	(8.39; 45.67)	(6.31; 38.87)	(0.02; 0.07)	(0.02; 0.11)	(0.02; 0.13)	(0.02; 0.08)
	05/01/2022 -	Median	1.52	2.19	3.92	1.47	0.01	0.01	0.01	0.01
	10/31/22	(Q1; Q3)	(0.63; 3.4)	(0.89; 4.85)	(1.47; 6.44)	(0.60; 3.37)	(0; 0.01)	(0.01; 0.02)	(0.01; 0.03)	(0.01; 0.03)
	12/01/2022 -	Median	1.06	1.58	2.46	0.86	0.01	0.01	0.01	0.01
	2/28/23	(Q1; Q3)	(0.54; 2.96)	(0.75; 4.27)	(1.31; 6.31)	(0.41; 2.09)	(0; 0.02)	(0.01; 0.03)	(0; 0.03)	(0.00; 0.01)
**Day 7**	12/01/2021 -	Median	5.72	9.62	20.58	14.15	0.04	0.06	0.08	0.04
	3/31/22	(Q1; Q3)	(2.66; 12.4)	(4.54; 23.1)	(9.7; 52.99)	(6.43; 40.09)	(0.02; 0.08)	(0.03; 0.12)	(0.03; 0.14)	(0.02; 0.09)
	05/01/2022 -	Median	1.48	2.84	4.48	1.57	0.01	0.01	0.01	0.02
	10/31/22	(Q1; Q3)	(0.62; 3.33)	(1.19; 6.75)	(1.67; 7.41)	(0.66; 3.73)	(0; 0.01)	(0.01; 0.03)	(0.01; 0.03)	(0.01; 0.03)
	12/01/2022 -	Median	0.98	2.05	2.83	0.88	0.01	0.01	0.01	0.01
	2/28/23	(Q1; Q3)	(0.5; 2.66)	(1; 5.72)	(1.52; 7.15)	(0.42; 2.17)	(0; 0.02)	(0.01; 0.03)	(0; 0.03)	(0.00; 0.01)

Table A6. County-Level Mean Absolute Error (MAE) and interquartile range (IQR) for weekly prediction.

			Full Model with X Input	Full Model with no X Input	Persistence model	ARIMA	Full Model with X Input	Full Model with no X Input	Persistence model	ARIMA
			Case				Death			
Week 1	12/01/2021 -	Median	91.57	139.09	138.36	113.08	0.41	0.52	0.5	0.43
	3/31/22	(Q1; Q3)	(43.44; 217.28)	(67.16; 365.43)	(67.94; 359.6)	(48.30; 300.89)	(0.18; 0.8)	(0.2; 0.94)	(0.19; 0.94)	(0.18; 0.64)
	05/01/2022 -	Median	21.54	23.49	30.48	23.14	0.1	0.11	0.1	0.14
	10/31/22	(Q1; Q3)	(8.32; 38)	(8.31; 42.9)	(11.25; 49.34)	(8.56; 39.50)	(0.04; 0.19)	(0.06; 0.27)	(0.05; 0.22)	(0.07; 0.28)
	12/01/2022 -	Median	15.29	16.7	19.38	14.81	0.06	0.11	0.06	0.08
	2/28/23	(Q1; Q3)	(7.42; 36.43)	(8.7; 38.95)	(10.13; 46.48)	(7.74; 42.53)	(0.03; 0.15)	(0.04; 0.25)	(0.03; 0.2)	(0.05; 0.15)
Week 2	12/01/2021 -	Median	93.14	140.17	267.22	259.31	0.38	0.53	0.8	0.78
	3/31/22	(Q1; Q3)	(44.36; 237.99)	(69.39; 367.08)	(126.11; 702.9)	(123.15; 731.75)	(0.16; 0.72)	(0.19; 0.9)	(0.36; 1.64)	(0.36; 1.30)
	05/01/2022 -	Median	21.81	28	38.22	41.1	0.1	0.1	0.2	0.26
	10/31/22	(Q1; Q3)	(8.33; 37.38)	(10.26; 48.16)	(16.04; 74.79)	(15.68; 76.25)	(0.04; 0.2)	(0.05; 0.22)	(0.1; 0.4)	(0.15; 0.53)
	12/01/2022 -	Median	14.66	18.62	26.91	25.62	0.08	0.07	0.12	0.14
	2/28/23	(Q1; Q3)	(7.21; 35.16)	(9.82; 43.56)	(15.42; 60.2)	(14.11; 69.12)	(0.03; 0.19)	(0.03; 0.21)	(0.05; 0.38)	(0.08; 0.26)
Week 3	12/01/2021 -	Median	96.86	140.33	374.63	402.9	0.37	0.5	1.11	1.05
	3/31/22	(Q1; Q3)	(47.17; 257.68)	(68.94; 359.12)	(176.18; 984.11)	(192.75; 1124.80)	(0.15; 0.65)	(0.2; 0.92)	(0.5; 2.33)	(0.53; 2.01)
	05/01/2022 -	Median	22.52	30.67	48.99	48.17	0.08	0.1	0.29	0.36
	10/31/22	(Q1; Q3)	(8.67; 38.52)	(11.41; 49.84)	(20.59; 92.61)	(21.12; 92.28)	(0.04; 0.18)	(0.05; 0.23)	(0.16; 0.58)	(0.21; 0.70)
	12/01/2022 -	Median	14.43	19.46	36.11	32.42	0.08	0.07	0.16	0.19
	2/28/23	(Q1; Q3)	(7.56; 35.71)	(10.21; 46.73)	(16.82; 81.82)	(15.35; 75.83)	(0.03; 0.17)	(0.03; 0.23)	(0.08; 0.5)	(0.12; 0.37)
Week 4	12/01/2021 -	Median	100.7	137.09	454.5	521.71	0.38	0.51	1.32	1.43
	3/31/22	(Q1; Q3)	(50.15; 288.72)	(67.62; 350.21)	(213.96; 1186.97)	(255.46; 1558.11)	(0.16; 0.64)	(0.21; 0.96)	(0.64; 2.93)	(0.70; 2.72)
	05/01/2022 -	Median	23.51	34.19	56.26	54.94	0.08	0.11	0.4	0.47
	10/31/22	(Q1; Q3)	(8.82; 40.61)	(12.33; 57.05)	(22.24; 120.7)	(23.59; 123.38)	(0.04; 0.18)	(0.05; 0.25)	(0.21; 0.76)	(0.25; 0.90)
	12/01/2022 -	Median	15.7	20.81	41.27	36.66	0.06	0.1	0.22	0.21
	2/28/23	(Q1; Q3)	(8.48; 38.48)	(10.91; 50.05)	(19.99; 95.48)	(17.75; 86.07)	(0.03; 0.15)	(0.04; 0.26)	(0.11; 0.57)	(0.14; 0.49)
	12/01/2021 -	Median	111.94	136.32	515.66	639.68	0.41	0.53	1.5	1.62

	3/31/22	(Q1; Q3)	(54.93; 318.45)	(68.42; 364.77)	(239.4; 1347.23)	(311.32; 1922.05)	(0.16; 0.67)	(0.21; 0.94)	(0.72; 3.58)	(0.84; 3.35)
**Week 5**	05/01/2022 - 10/31/22	Median	26.32	37.36	61.92	64.08	0.09	0.11	0.49	0.59
		(Q1; Q3)	(9.7; 45.74)	(13.56; 60.19)	(24.54; 141.82)	(25.96; 146.51)	(0.04; 0.2)	(0.05; 0.25)	(0.28; 0.95)	(0.29; 1.09)
	12/01/2022 - 2/28/23	Median	18.02	22.23	46.08	42.55	0.06	0.08	0.26	0.25
		(Q1; Q3)	(10.05; 47.94)	(12.05; 55.14)	(22.2; 109.48)	(19.76; 101.51)	(0.03; 0.16)	(0.03; 0.24)	(0.13; 0.63)	(0.16; 0.57)

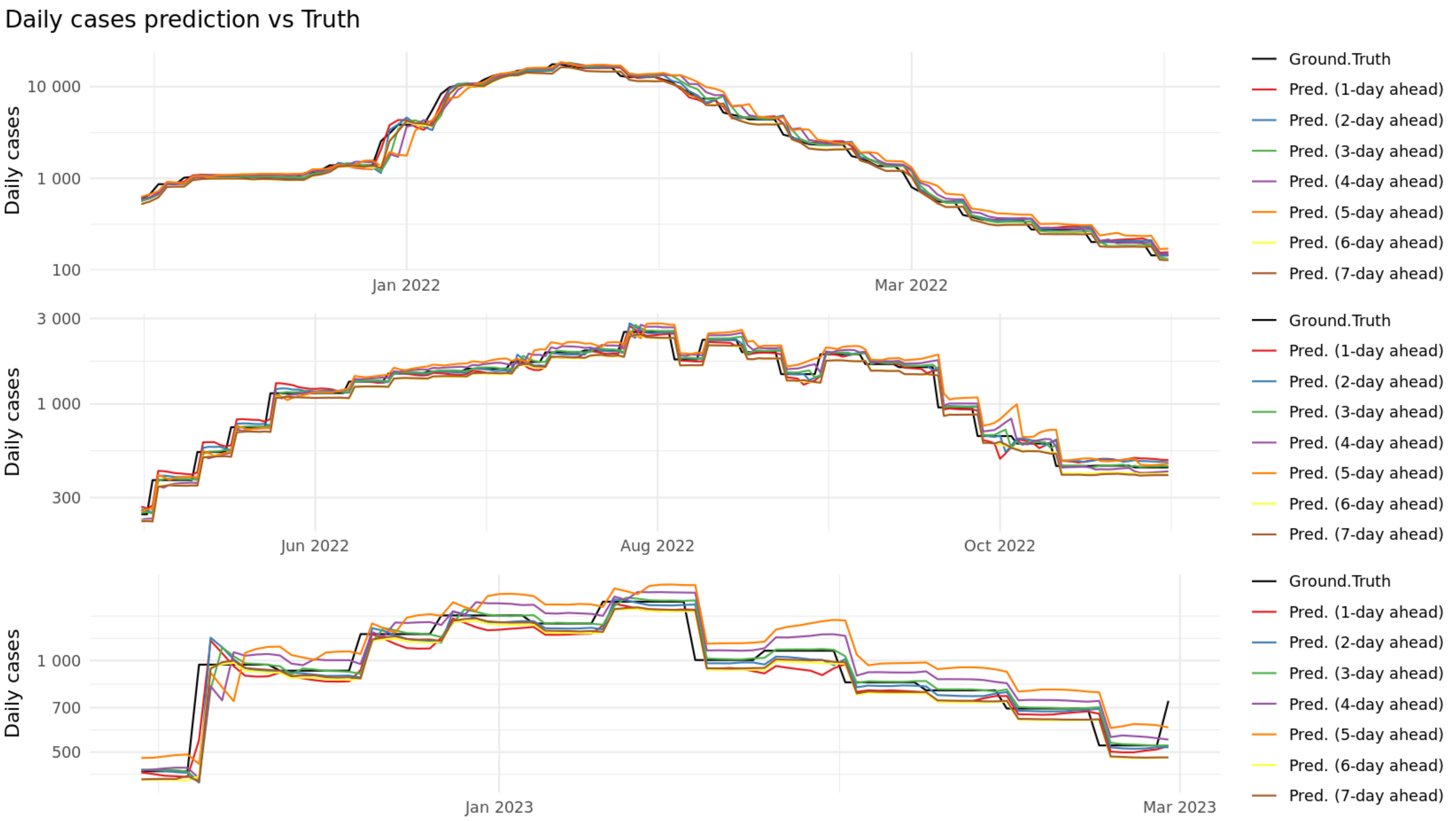

Figure A1. Comparison of total daily predicted and actual values of Covid-19 positive cases.

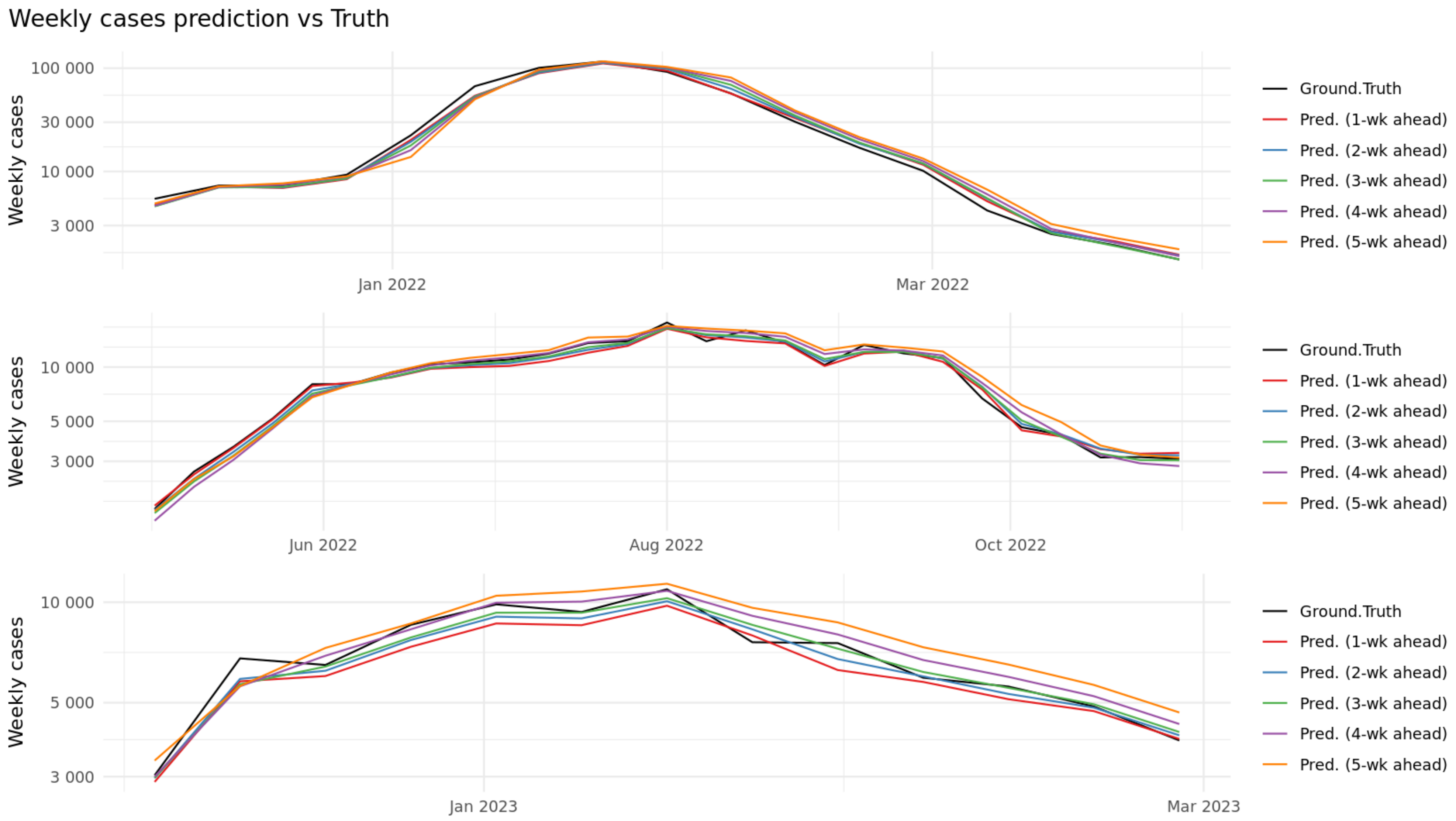

Figure A2. Comparison of total weekly predicted and actual values of Covid-19 positive cases.

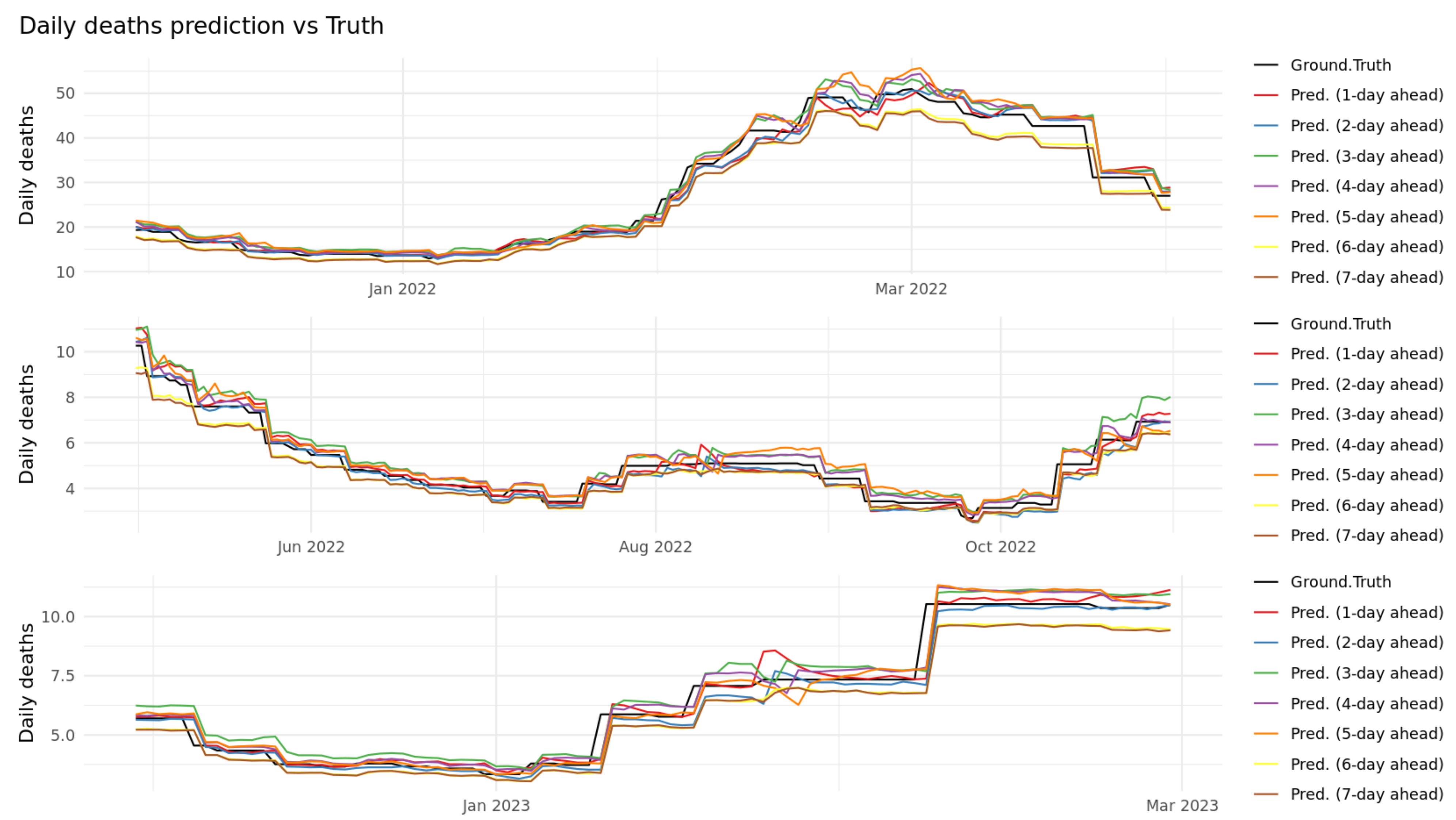

Figure A3. Comparison of total daily predicted and actual values of Covid-19 deaths.

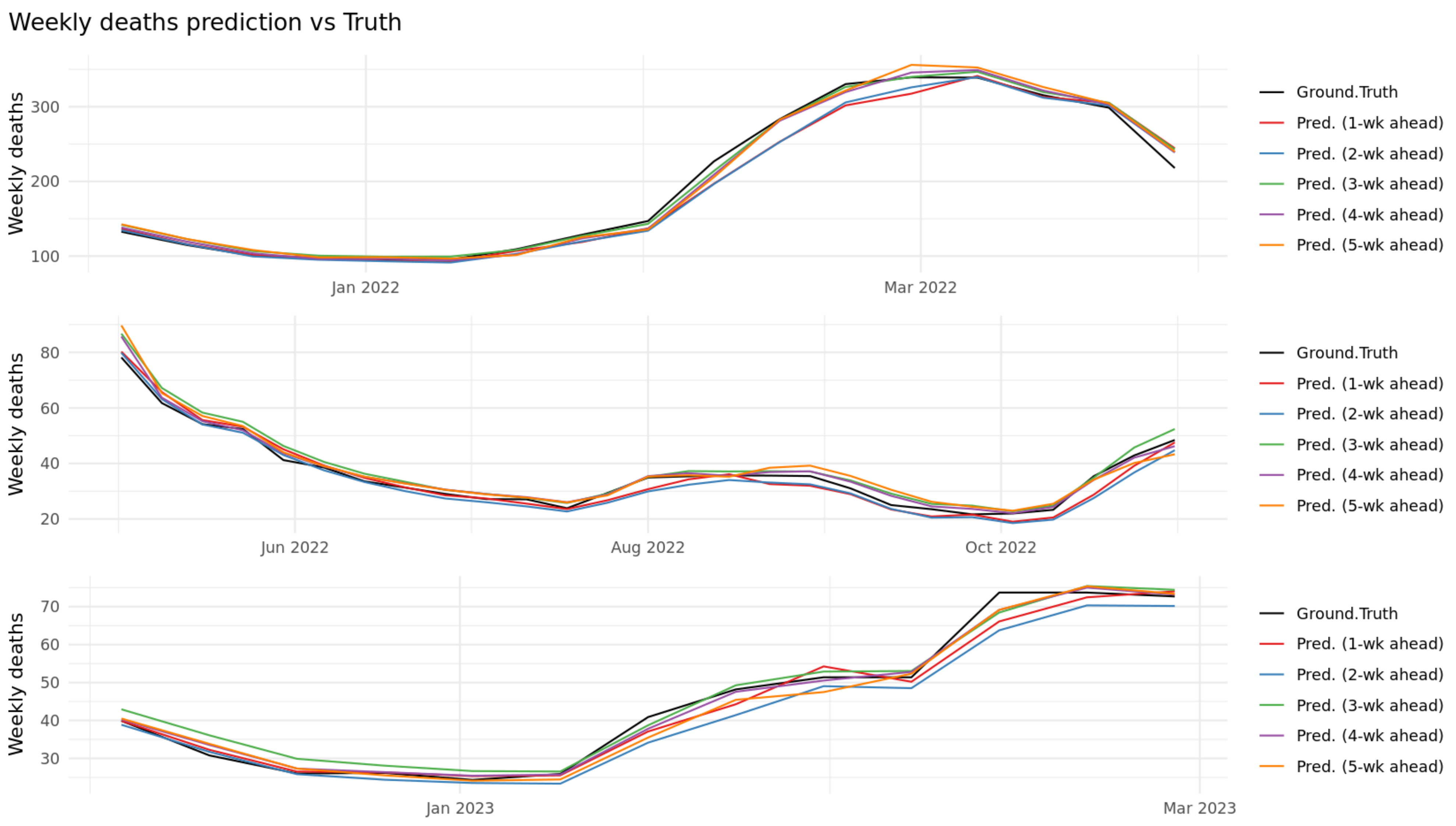

Figure A4. Comparison of total weekly predicted and actual values of Covid-19 death.

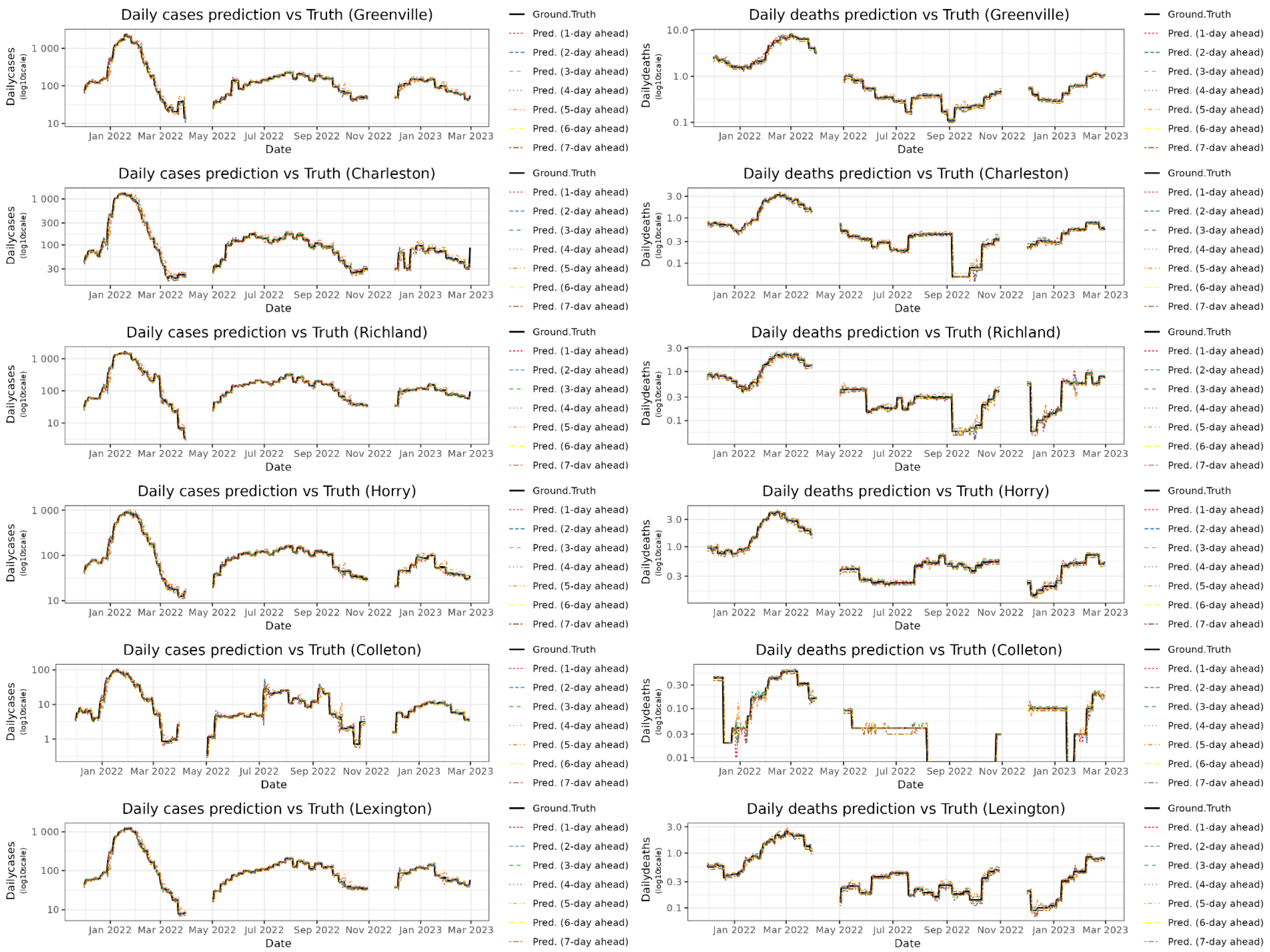

Figure A5. Comparison of daily predicted and actual values of Covid-19 positive cases and deaths for several representative counties. Left: daily case prediction, Right: daily death prediction.

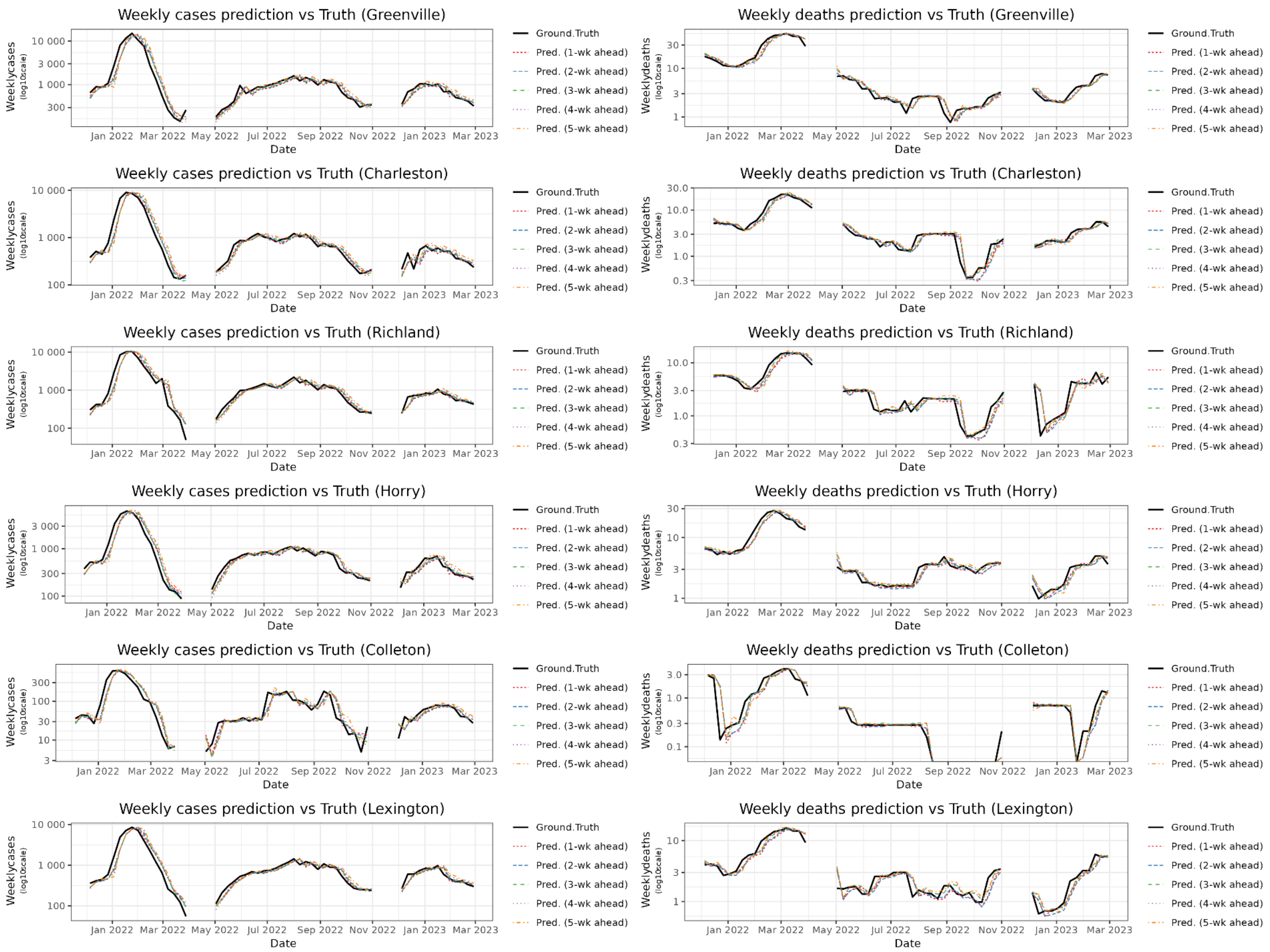

Figure A6. Comparison of weekly predicted and actual values of Covid-19 positive cases and deaths for several representative counties. Left: daily case prediction, Right: daily death prediction.
